# Reversal by RARα agonist Am580 of c-Myc-induced imbalance in RARα/RARγ expression during MMTV-Myc tumorigenesis

**DOI:** 10.1186/bcr3247

**Published:** 2012-08-24

**Authors:** Almudena Bosch, Silvina P Bertran, Yongke Lu, Avalon Garcia, Alexis M Jones, Marcia I Dawson, Eduardo F Farias

**Affiliations:** 1 Mount Sinai School of Medicine, Department of Medicine, The Tisch Cancer Institute, Division of Hematology and Medical Oncology, One Gustave L Levy Place, New York, NY 10029, USA; 2 Cancer Center, Sanford-Burnham Medical Research Institute, 10901, North Torrey Pines Road, La Jolla, CA 92037, USA

## Abstract

**Introduction:**

Retinoic acid signaling plays key roles in embryonic development and in maintaining the differentiated status of adult tissues. Recently, the nuclear retinoic acid receptor (RAR) isotypes α, β and γ were found to play specific functions in the expansion and differentiation of the stem compartments of various tissues. For instance, RARγ appears to be involved in stem cell compartment expansion, while RARα and RARβ are implicated in the subsequent cell differentiation. We found that over-expressing *c-Myc *in normal mouse mammary epithelium and in a *c-Myc*-driven transgenic model of mammary cancer, disrupts the balance between RARγ and RARα/β in favor of RARγ.

**Methods:**

The effects of *c-Myc *on *RAR *isotype expression were evaluated in normal mouse mammary epithelium, mammary tumor cells obtained from the MMTV-Myc transgenic mouse model as well as human normal immortalized breast epithelial and breast cancer cell lines. The *in vivo *effect of the RARα-selective agonist 4-[(5,6,7,8-tetrahydro-5,5,8,8-tetramethyl-2-naphthyl)carboxamido]benzoic acid (Am580) was examined in the MMTV-Myc mouse model of mammary tumorigenesis.

**Results:**

Modulation of the *RARα/β *to *RARγ *expression in mammary glands of normal mice, oncomice, and human mammary cell lines through the alteration of RAR-target gene expression affected cell proliferation, survival and tumor growth. Treatment of MMTV-Myc mice with the RARα-selective agonist Am580 led to significant inhibition of mammary tumor growth (~90%, *P*<0.001), lung metastasis (*P*<0.01) and extended tumor latency in 63% of mice. Immunocytochemical analysis showed that in these mice, RARα responsive genes such as Cyp26A1, E-cadherin, cellular retinol-binding protein 1 (CRBP1) and p27, were up-regulated. In contrast, the mammary gland tumors of mice that responded poorly to Am580 treatment (37%) expressed significantly higher levels of RARγ. *In vitro *experiments indicated that the rise in RARγ was functionally linked to promotion of tumor growth and inhibition of differentiation. Thus, activation of the RARα pathway is linked to tumor growth inhibition, differentiation and cell death.

**Conclusions:**

The functional consequence of the interplay between *c-Myc *oncogene expression and the *RARγ *to *RARα/β *balance suggests that prevalence of *RARγ *over-*RARα/β *expression levels in breast cancer accompanied by *c-Myc *amplification or over-expression in breast cancer should be predictive of response to treatment with RARα-isotype-specific agonists and warrant monitoring during clinical trials.

See related editorial by Garattini et al http://breast-cancer-research.com/content/14/5/111

## Introduction

The retinoic acid (RA) nuclear receptor isotypes retinoic acid receptor (RAR)α, RARβ and RARγ have many overlapping as well as unique functions [1-4]. The RARs belong to the steroid/thyroid hormone superfamily of ligand-dependent transcription factors [5-8], bind both all-trans retinoic acid (ATRA) and its isomer, 9-cis RA, and form heterodimers with the retinoid × receptor isotypes (RXRs α-γ) [9]. ATRA functions as a pan-agonist of all three RAR isotypes thereby playing crucial roles in embryonic morphogenesis, cell differentiation and maintenance of adult epithelia [10,11]. These findings together with preclinical, epidemiological and clinical observations [12] have prompted extensive inquiries into ATRA's potential use as an anti-tumor agent. Despite its demonstrated anti-tumor activity *in vitro *and in a limited number of cancer models [13-21] and the highly positive response observed in acute promyelocytic leukemia patients [22-24], clinical trials using ATRA as a treatment for solid tumors have produced disappointing results overall [25-29].

Although the RAR isotypes display overlapping functions as evidenced by their ability to modulate common target genes [30,31], Husman *et al*. [32] described evidence of antagonism between RAR isotypes. Specifically, RARγ1 inhibited functions of other RAR isotypes. In addition, different RAR isotypes can transcribe the same target gene with different efficiencies, with transcription further modulated by their phosphorylation status. Moreover, interactions between isotypes are dynamic and affected by both intracellular and extracellular environments such as changes in cell signaling induced by oncogenic stress and global kinase activity [33].

Studies of the RAR isotypes and their roles in mammary development and breast cancer provide the first clues to the unique activities that certain RAR isotypes have and suggest that certain isotype-selective retinoids may have therapeutic potential against breast cancer. It was shown that specific activation of RARα induces the expression of RARβ, which is required for normal tissue differentiation [10,11]. Similarly, activation of RARα also induced the expression of the cellular retinol-binding protein-1 (CRBP1), a key retinol chaperone in the cellular metabolism of retinol to ATRA, and maintained the differentiated status of the mature epithelial phenotype [34-37]. In contrast, the RARγ isotype had pro-tumorigenic activity in liver cancer models [38], and its activation stimulated breast cancer cell proliferation [39]. On this basis, we tested whether the unique functions of these RAR isotypes could be translated into an effective approach to anti-tumor therapy. To achieve this goal we selected the synthetic retinoid Am580, which is reported to be an RARα-selective transcriptional agonist [40] that does not activate RARγ [40]. Previously, we showed that in the MMTV-Wnt1 and MMTV-Neu transgenic mouse models of breast cancer, in which the oncogene expression is driven by the mouse mammary tumor virus promoter (MMTV), treatment with Am580 [40] significantly prolonged tumor-free survival and impaired tumor growth [39]. In contrast, treatment of MMTV-Neu transgenic mice with the RAR isotype pan-agonist ATRA, which also activates RARγ, promoted tumor growth [41]. Most importantly, these results further demonstrated the reciprocal relationship between RARα and RARγ, whereby direct inhibition of RARγ activity either by a specific RARγ antagonist or by indirect inhibition by ligand-mediated RARα activation leading to down-regulation of RARγ, allowed expression of RARα and its target genes, *RARβ *and *CRBP1*. Blocking RARγ while simultaneously activating RARα, strongly impinged on oncogene-induced growth pathways to attenuate the transforming potential of both *Neu *and *Wnt1 *oncogenes [39,42,43].

This newly discovered cross-regulation of RAR isotypes, coupled with the cancer-promoting role of RARγ and anti-cancer role of RARα, prompted us to investigate their roles in the MMTV-Myc mammary cancer mouse model, in which parous females develop mammary carcinomas with 100% incidence following a latency period of several months. This model is representative of about 30% of human breast cancer cases in which *c-Myc *is amplified and/or over-expressed [44]. The *c-myc *gene is often over-expressed in tumors having mutations in the *BRCA1 *gene [44]. By forming a heterodimer with Max, c-Myc transactivates several proliferation-related genes and consequently prevents Max from forming a Mad/Max heterodimer [45,46] that represses transcription of cell-cycle/growth arrest genes such as *p21^waf1/CIP1^*, *p27^kip1 ^*and *gadd45*, and the angiogenesis inhibitor thrombospondin-1 [47-49], which are also RARα regulated genes.

Here, we examined whether *c-Myc *over-expression affected the expression of RAR isotypes and their target genes in normal mouse mammary gland epithelial cells. The goal was to determine whether *RARγ *expression was enhanced and whether this increase affected c-Myc-induced tumor growth. We also evaluated whether specific activation of RARα by Am580 had anti-tumor effects in c-Myc-induced tumorigenesis.

Overall, our results bring new insights to our understanding of the effect of the *c-Myc *oncogene on RAR isotype expression, c-Myc/RAR isotype reciprocal relationships, and the novel tumor-promoting role of RARγ. We propose that the characterization of RAR expression in breast cancer will identify patients that would benefit from RAR isotype-selective retinoid treatment.

## Materials and methods

### Antibodies

Antibody sources were as follows: anti-p27 and anti-E-cadherin (BD Transduction Labs, Hoboken, NJ, USA); anti-CYP26A1 and anti-CRBP1 (Santa Cruz Biotechnology, CA); anti-RARα, -RARβ and -RARγ (Abcam, Cambridge, MA, USA), anti-Akt, anti-pAkt anti-pErk, anti-Erk and anti-pRB (Cell Signaling, Beverly, MA, USA); anti-GAPDH (Calbiochem, Gibbstown, NJ, USA); anti-tubulin (Sigma Diagnostics, St. Louis, MO, USA).

### Immunoblotting

Tumor samples were mechanically homogenized in radioimmunoprecipitation assay (RIPA) buffer (1% Triton X-100, 10 mM Tris, pH 8, 140 mM NaCl, and 0.1% SDS). Primary cultures derived from wild type FVB mice mammary gland epithelium were washed in PBS, pH 7.4, and lysed with RIPA buffer. Immunoblotting was performed following standard procedures as described previously [39].

### *In vivo *studies

Three-month-old uniparous MMTV-Myc female mice (NCI Frederick Mouse Repository, Frederick, MD, USA) (30 mice/group) were fed with 0.3 mg/kg/day of the RARa agonist Am580 (4-[(5,6,7,8-tetrahydro-5,5,8,8-tetramethyl-2-naphthyl)carboxamido]benzoic acid), which was kindly provided by Dr K Shudo (Research Foundation Itsuu Laboratory, Tokyo, Japan). Am580 was mixed into their regular diet by the vendor (Purina 5053, Richmond, IN, USA). Food consumption was measured to calculate the amount of Am580 to be added to the diet to achieve the daily dose as described previously [39]. Regular diet was used as the control. Because the objective was to study the effect of Am580 on tumor initiation and development, mice that developed tumors within the first month were removed from the study on the assumption that their tumors had developed before treatment began. Mice were palpated twice weekly and the onset of tumor development was recorded. Once palpable, the tumor sizes were measured weekly in two dimensions and volumes calculated using the equation Vol = Dxd^2^/2 (where D = major diameter and d = minor diameter). Tumor-free survival was calculated from Kaplan-Meier curves, and statistical significance was determined using the log-rank test for survival and the *t*-test for tumor growth. Metastasis dissemination was evaluated by dissecting the lungs from euthanized mice and inspecting the Bouin-fixed (Sigma, St. Louis, MO, USA) lung surface for lesions using a stereoscope (Nikon SMZ800 stereoscope X3 to X5). For xenograft experiments, 8-week-old syngenic FVB mice were used (NCI Frederick Mouse Repository). Cells or tumor fragments were inoculated into the mammary fat pad of the inguinal mammary glands (gland numbers 4 and 8) under soft anesthesia and analgesia in accordance with the Institutional Animal Care and Use Committee (IACUC) guidelines.

### *In Vivo *protocol approval

Protocols designed and used in the *in vivo *experiments were approved by the Mount Sinai School of Medicine (MSSM) IACUC and conducted following its guidelines.

### Immunohistochemistry

Tumor samples were fixed in 10% buffered formalin for 24 h, transferred to 70% ethanol and kept at 4ºC until use. Sections were prepared from eight tumors per group, subjected to standard antigen retrieval and incubated with primary antibody overnight at 4ºC. Sections were processed using the VectaStain ABC Elite Kit (Vector Laboratories, Burlingame, CA, USA), signals were detected using the Metal Enhanced DAB Substrate Kit (Pierce Laboratories, Rockford, IL, USA) and sections counterstained with Harris Hematoxylin Solution (Sigma Diagnostics).

### Cell lines, culture conditions and plasmid transfection

After euthanasia by CO_2 _overdose, tumors from untreated female MMTV-Myc mice and mammary glands from wild-type-FVB female mice were removed by dissection and minced into fragments, which were then digested with 5 ml of 1.5 mg/ml collagenase (Sigma, St. Louis, MO, USA) in PBS containing 25 mg/ml BSA (Sigma), 100 mM Ca^2+ ^and 100 mM Mg^2+ ^per approximate 500 mg of tissue at 37ºC for 30 to 45 minutes with gentle agitation. Cells were maintained in DMEM-F12 (Cellgro, Manassas, VA, USA) containing 5% (MMTV-Myc cells) or 10% (wt-FVB cells) fetal bovine serum (FBS) and 4 μg/ml insulin (Sigma Diagnostics). Immortalized nontumorigenic human MCF-10A breast epithelial cells, which were a generous gift from Dr J Brugge (Department of Cell Biology, Harvard Medical School, Boston, MA, USA), were maintained as described by Debnath *et al*. [50]. Human breast cancer MCF-7 (ER^+^) and MDA-MB-231 (ER^-^) cells were obtained from the American Tissue Culture Collection (ATCC, Manassas, VA, USA). Both cell lines were propagated using ATCC protocols. FVB or Myc cells were transfected with either pSG5-empty, pSG5-*RARγ*, pSG5-m*CRBP1 *(kindly provided by Dr Chambon, IGBMC, Strasbourg, France), pcDNA3, or pcDNA3-h-*c-Myc *(Addgene, Ricci *et al*. [51]) vectors using Lipofectamine 2000 (Invitrogen, Carlsbad, CA, USA) in Opti-MEM (Gibco, Carlsbad, CA, USA). At 24 h after transfection, cells were treated for 16 h with 1 μM ATRA (Sigma, St. Louis, MO, USA) in dimethyl sulfoxide (DMSO) (0.01% final concentration; Sigma Diagnostics).

### siRNA transfection using Lipofectamine RNAiMAX

MCF-10A, MCF-7 and MDA-MB-231 cells were plated and grown to 30 to 40% confluence at 24 h. One hour prior to transfection the medium was replaced with 1X Opti-MEM reduced serum medium (Gibco, Carlsbad, CA, USA). Anti-*RARγ *siRNA and a scramble control siRNA sequence (Sigma-Aldrich; seq1 #SASI_Hs01_00012455, seq2 #SASI_Hs01_00012456, Scramble seq #SIC001) were transfected according to the Invitrogen protocol using Lipofectamine RNAiMax reagent (Invitrogen catalogue number 13778-075, Grand Island, NY, USA).

### Cell lines and 3D cultures

Primary Myc cell suspensions obtained after a 45-minute collagenase digestion (1.5 mg/ml collagenase and 25 mg/ml BSA in PBS plus 100 mM Ca^2+ ^and 100 mM Mg^2+^) of MMTV-Myc tumor fragments were grown in DMEM/F12 medium supplemented with 5% horse serum, 100 ng/ml cholera toxin, 5 mg/ml insulin, 0.5 mg/ml hydrocortisone, 1% Pen/Strep, 1% glutamine (Gibco), 1% non-essential amino acids (Invitrogen) and 20 ng/ml EGF (PeproTech, Rock Hill, NJ, USA). Cells were transfected with shRARγ(2 μg) in 35-mm dishes, following the manufacturer's instructions (Open Biosystems, Huntsville, AL, USA) and seeded (3 × 10^3^/well) in quadruplicate onto Matrigel^® ^(BD Bioscience, San Jose, CA, USA) beds in 8-well culture slides (BD Bioscience, Bedford, MA, USA) to prepare three-dimensional cultures as described by Debnath *et al*. [50]. The media was changed every 48 h for 8 consecutive days. An additional shRARγ transfection was done at day 4 to maintain *RARγ *knockdown. Colony morphology was determined by phase-contrast microscopy. RARs were silenced using shRNAs from Open Biosystems (Open Biosystems, Huntsville, AL, USA) shRARα (Oligo ID: V2MM_6881) , shRARβ (Oligo ID: V2HS_239292) or shRARγ (Oligo ID: V2MM_62330). Scrambled shRNA (Open Biosystems, Huntsville, AL, USA) was used as the control.

### Proliferation assay

Primary Myc cells (2 × 10^4^) were seeded in triplicate in 6-well culture dishes and allowed to attach overnight. They were then washed with culture medium and treated with the RARγ antagonist SR11253 (2-(4-carboxyphenyl)-2-(5,6,7,8-tetrahydro-5,5,8,8-tetramethyl-2-naphthyl)-1,3-dithiolane) at increasing doses (10, 50 and 250 nM) or with DMSO (0.001% final concentration) alone, and then detached with 0.05% trypsin (Gibco) and counted every 24 h for 4 days. Statistical significance was determined by *t*-test. Following the same protocol described above for Myc, MCF-10A, MCF-7 and MDA-MB-231 cells were treated with 1 μM ATRA (RAR pan-agonist), 200 nM Am580 (RARα agonist), 100 nM CD437 (the RARγ/β agonist 6-[3-(1-adamantyl)-4-hydroxyphenyl]-2-naphthalenecarboxylic acid, AHPN) (Sigma), 30 nM BMS961 (the RARγ agonist 3-fluoro-4-[[2-hydroxy-2-(5,5,8,8-tetramethyl-5,6,7,8-tetrahydro-2-naphthalenyl)acetyl]amino]benzoic acid) (Tocris, Bristol, UK) and SR11253 or DMSO (0.001% final concentration) alone as the control.

### Real-time PCR

Total RNA from FVB mammary gland epithelial cells was isolated using the RNeasy mini kit (Qiagen, Valencia, CA, USA) and reverse-transcribed using the iScript cDNA synthesis kit (Bio-Rad, Philadelphia, PA, USA). cDNA was amplified by real-time PCR using an iQ5 Real-Time PCR detection systems kit (Bio-Rad) and SYBR green PCR master mix (Applied Biosystems, Carlsbad, CA, USA). Primer sequences were: *RARγ*1-sense: 5'-TGG GGC CTG GAT CTG GTT AC-3', RARγ1-antisense: 5'-TTC ACA GGA GCT GAC CCC AT; *RARγ2*-sense: 5'-GCC GGG TCG CGA TGT ACG AC and *RARγ2*-antisense: 5'- TTC ACA GGA GCT GAC CC CAT; *RARβ2*-sense: 5'-ATG GAG TTC GTG GAC TTT TCT GTG-3' and *RARβ2*-antisense: 5'-CTC GCA GGC ACT GAC GCC AT-3'; *CRBP1*-sense: 5'-ACG GGT ACT GGA AGA TGC TG-3' and *CRBP1*-antisense: 5'-CCA TCC TGC ACG ATC TCT TT-3'; Hmq-*c-Myc*-sense: 5'-AGC GAC TCT GAG GAG GAA CA-3' and Hmq-*c-Myc*-antisense: 5'-AGT GGG CTG TGA GGA GGT TT-3'; *GAPDH*-sense: 5'-CGT AGA CAA AAT GGT GAA GG-3' and *GAPDH*-antisense: 5'-GAC TCC ACG ACA TAC TCA GC-3'.

### Chromatin immunoprecipitation assay

Cells from FVB mammary gland epithelium were isolated as described above and seeded in 140-mm plates pre-coated with Matrigel^® ^(BD Bioscience, San Jose, CA, USA). At 70% confluency, cells were transfected with pSG5 or pSG5-*RARγ *(10 μg) using Lipofectamine 2000 in Opti-MEM, 24 h after transfection, cells were treated with 1 μM ATRA for 16 h, and then crosslinked using 1% formaldehyde. Cell nuclei were isolated using hypertonic buffer A (150 mM NaCl, 50 mM Tris-HCl, pH 8, 1% NP40, 1% sodium deoxycholate, 0.5% SDS, and 2 mM EDTA), centrifuged, lysed in SDS lysis buffer (50 mM Tris, pH 8,10 mM EDTA, 1% SDS), and then sonicated. Chromatin from the nuclear fraction obtained was pre-cleared with protein G agarose/sperm salmon (Millipore, Billerica, MA, USA) and irrelevant immunoglobulin G (IgG). RARγ was immunoprecipitated overnight at 4°C, and immunoprecipitates were incubated with protein G agarose/sperm salmon for 2 h at 4°C. Immunoprecipitates were washed and eluted with elution buffer (100 mM Na_2_CO_3 _and 1% SDS) from agarose beads and incubated in 5 M NaCl at 65°C overnight. DNA was isolated by digestion with proteinase K, treatment with RNAse A, and purification using the QIAquick DNA purification kit (Qiagen, Valencia, CA, USA). Primers for the detection of the RA response element (RARE) sequence of *CRBP1 *were designed based on Smith *et al*. [52]: *CRBP1*-sense 5'-CTT GCC TAC CCT GAT GGT GT-3' and *CRBP1*-antisense: 5'-CCC TTC TCA CCT GCT ACC TG-3'. As a control, nonspecific primers were designed using 9000-bp upstream of the *CRBP1 *promoter; nonspecific-sense: 5'-GCA AGA CTG CTT GCT CTC CT-3' and nonspecific-antisense: 5'-AAC ACA TCG TGG GTG GTC TT-3'.

## Results

### c-Myc up-regulates *RARγ *expression leading to down-regulation of *RARα *target gene, *CRBP1*

**Figure 1:**
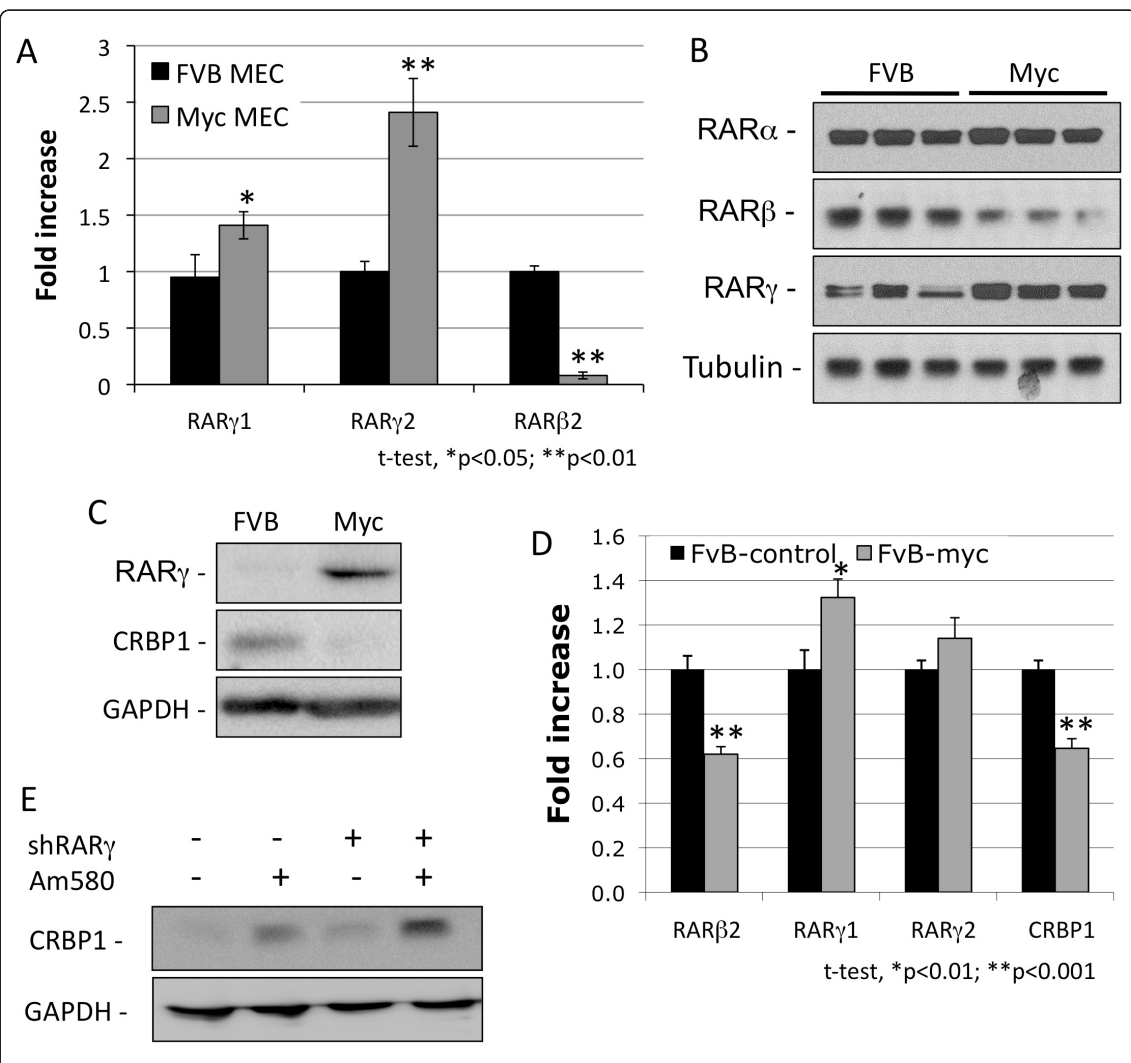
**Retinoic acid receptor (RAR)γ cooperates in Myc-induced tumorigenesis and down-regulation of *RARβ2 *and *CRBP1***. **A**) Quantitative (Q)-PCR analysis of *RARγ1*, γ2 and β2 expression in normal (FVB mammary epithelial cells, MEC) and hyperplastic (Myc MEC) mammary epithelium. **B**) Western blot analysis of the RAR isotypes in FVB mammary gland epithelium and MMTV-Myc tumors (three primary cultures/experimental group). **C**) Western blot analysis of RARγ and CRBP1 in Myc tumors and in normal FVB mammary epithelium. **D**) Q-PCR analysis of *RARβ2*, *RARγ1*, *RARγ2 *and *CRBP1 *expression in FVB cells transfected with a vector control or *c-Myc*. **E**) Induction of CRBP1 protein by Am580. Primary cultured Myc MECs or those stably transfected with shRARγ (Figure S1 in Additional file 1) were treated with 200 nM Am580 for 48 h. Glyceraldehyde-3-phosphate dehydrogenase (GAPDH) protein was used as a loading control.

We hypothesized that over-expression of *c-Myc *in the mammary epithelium of MMTV-Myc transgenic mice leads to up-regulation of *RARγ *and determine if this up-regulation happens before the development of palpable tumors. To test this hypothesis, we compared *RARγ *expression in mammary gland epithelium isolated from 15-week-old virgin MMTV-Myc female mice, at which point the mammary epithelium is already hyperplastic, to that of primary mammary epithelium from wt-FVB female mice of the same age. Quantitative (Q)-PCR results (Figure 1A) revealed that compared to normal epithelium, the MMTV-Myc mouse hyperplastic epithelium had elevated levels of *RARγ1 *and γ2 mRNA and reduced *RARβ2*, which is commonly silenced in cancer [53]. Western blot analysis confirmed that compared to primary cells cultured from normal FVB mammary epithelium, Myc tumors had elevated RARγ but reduced RARβ protein levels, whereas RARα levels remained unchanged (Figure 1B). Moreover, the elevation in the level of RARγ protein in c-Myc over-expressing cells compared to that in normal FVB cells, appeared to correlate with the reduction in the level of CRBP1 protein (Figure 1C).

To determine whether modulation of *RARγ *and *CRBP1 *were functionally linked to *c-Myc *expression, primary cultures of FVB mammary epithelial cells were transfected with *c-Myc *or a control vector (c-Myc expression is shown in Figure S1A in Additional file 1) and for the transfected cells were evaluated for the expression of *RARγ *and that of the RARα target genes *CRBP1 *and *RARβ *[5-8]. Forced expression of *c-Myc *specifically elevates *RARγ1 *mRNA relative to the vector control, but not that of *RARγ2 *(Figure 1D). Knockdown of *RARγ1 *in primary Myc cells using shRARγ1 (Figure S1B in Additional file 1) followed by Am580 treatment resulted in an even higher level of *CRBP1 *expression (Figure 1E), showing that in these cells RARγ has a repressive effect on the RARα target gene *CRBP1*.

These results suggest that when the RARγ ligand is absent or present only at low physiologic concentration, *RARγ *over-expression represses *CRBP1 *expression, but this effect is independent of its canonical activation.

### RARγ has a pro-tumorigenic function in c-Myc-induced tumorigenesis

**Figure 2:**
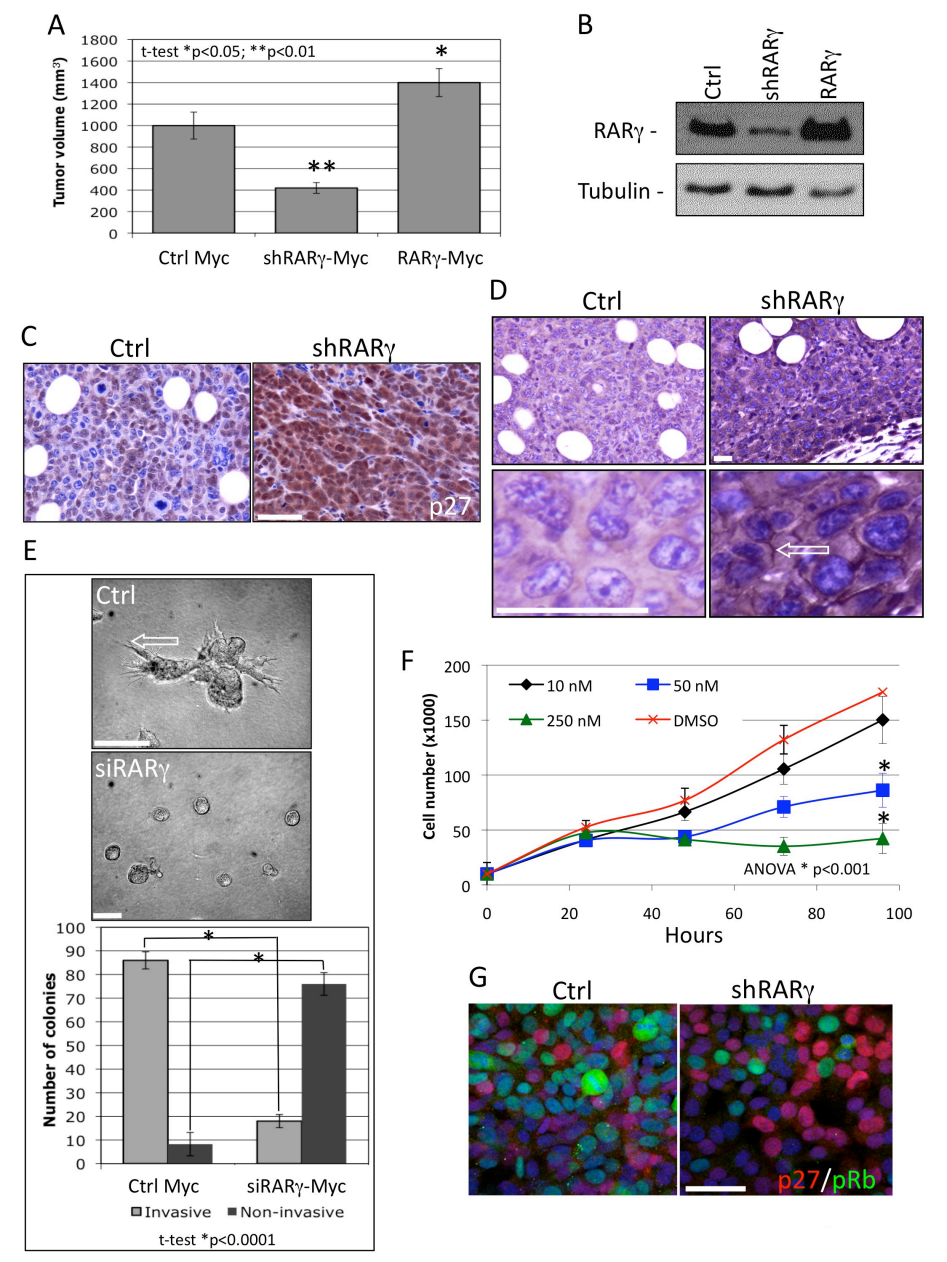
**Retinoic acid receptor (RAR)γ modulates tumor cell growth, differentiation and morphology in MMTV-Myc xenografts in FVB mice**. **A**) Primary cells from MMTV-Myc tumors were isolated and cultured. They were then transfected with the control vector pSG5 or a vector tethered to shRARγ or pSG5-*RARγ*, shRARγ or pSG5-*RARγ *plasmids. Transfected cells (0.5 × 10^6^) were inoculated orthotopically into the abdominal mammary gland fat pad (gland number 4) of 10 FVB female mice, whereas their contralateral fat pad was inoculated with pSG5-transfected cells to serve as an internal control. Tumor volume shown is after 15 days of *in vivo *growth. Results shown are representative of two independent experiments. **B**) Immunoblotting of tumor cell lysates with anti-RARγ antibody showing the levels of RARγ in the shRARγ and pSG5-*RARγ*-transfected Myc cells. Immunohistochemical analysis of five sections per group of tumors derived from the shRARγ-transfected cells compared to the vector control cells (**C **and **D**). **C**) Expression of cell growth-arrest marker p27 (brown); space bar = 100 um. **D**) Expression of differentiation marker E-cadherin. Plasma membrane-associated E-cadherin was detected in the shRARγ tumors (open arrow); space bar = 20 um. **E**) Morphologic analysis of Myc cells cultured under three-dimensional conditions in Matrigel^® ^(4 wells/group); bar = 100 μm. The number of colonies exhibiting the invasive (*t*-test *P *< 0.0001, light-gray bars) and noninvasive (*t*-test *P *< 0.0001, dark-gray bars) phenotype was quantified. **F**) RARγ antagonist SR11253 inhibits Myc tumor cell proliferation in a dose-dependent manner. Proliferating Myc cells were treated with increasing concentrations of SR11253 as shown. **G**) Confocal immunofluorescence for p27 (cell arrest marker, red) and pRB (proliferation marker, green) in Myc cells transfected with the shRARγ construct and in vector alone-transfected control cells; bar = 50 μm. Panels **E**, **F **and **G **are representative of three independent experiments.

Our results suggest that RARγ is involved in the repression of RARβ and CRBP1 expression, both of which have a tumor-suppressive function [54]. To determine whether RARγ has a pro-tumorigenic function in the context of c-Myc-driven transformation, *RARγ *was either stably knocked down or over-expressed in primary Myc cells and inoculated orthotopically into the mammary fat pad of syngeneic FVB mice. After 20 days, the tumors formed by *RARγ *null myc cells displayed a 60% reduction in size in comparison to those formed by Myc-control cells. The Myc cells in which *RARγ *was over-expressed produced approximately 40% larger tumors (Figure 2A and 2B). These results allude to the fact that *RARγ *is likely to confer pro-tumorigenic activity.

Immunohistological analysis of tumor sections isolated from mice implanted with shRARγ-transfected Myc cells showed increased expression of p27 (Figure 2C), which is a marker of cell-cycle arrest [55], and membrane-associated E-cadherin (Figure 2D arrow), which is a marker of epithelial cell differentiation [56].

Given the pro-tumorigenic activity of RARγ, we examined the effect of RARγ knockdown on tumor invasiveness by generating three-dimensional cultures. In this approach, Myc cells were oligofected with siRARγ both before being plated in Matrigel^® ^as described by Debnath *et al*. [50] and then again on day 4. The colonies were then cultured for 8 days before the size and morphology of the resulting colonies were determined. Colonies formed by control cells transfected by vector alone were large (>200 um) and invasive, with long matrix-penetrating spikes. In contrast, the siRARγ-transfected cells developed only small (around 100 μm), smooth, rounded acinar-like colonies. The difference in invasiveness between control and siRARγ-transfected cells was quantified by counting 100 colonies per group (n = 4 wells/group) and was found to be highly statistically significant (Figure 2E).

In addition to knocking down *RARγ*, we also explored the effect of pharmacologic inhibition of its activity using the RARγ antagonist SR11253 [57,58] in Myc cells *in vitro*. SR11253 treatment reduced Myc cell proliferation in a dose-dependent manner (IC_50 _value = 50 nM after 96 h) (Figure 2F). The anti-proliferative effect achieved by reducing the expression of *RARγ *coincided with up-regulation of p27 and down-regulation of pRb, as shown by confocal immunofluorescence (Figure 2G).

These results show that *c-Myc *over-expression increases the level of RARγ. Up-regulation of RARγ led to proliferation and invasiveness.

### RARγ supports proliferation and survival of human breast cell lines

**Figure 3:**
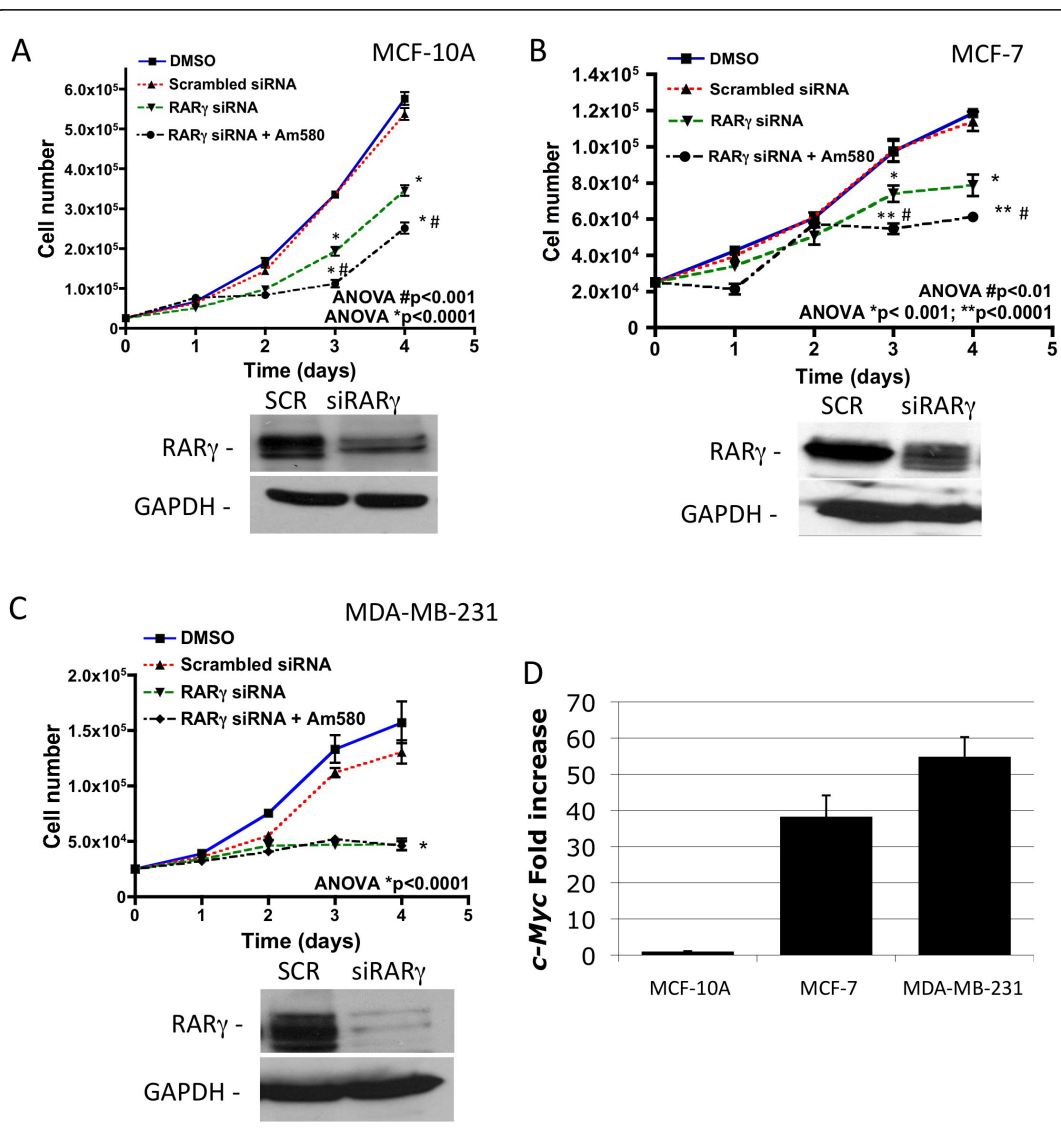
**Cell proliferation assays of human cell lines**. **A**-**C**). Effect of knockdown of retinoic acid receptor (RAR)γ on proliferation of human MCF-10A immortalized, non-tumorigenic mammary epithelial cells (**A**), MCF-7 breast cancer cells (**B**) and MDA-MB-231 breast cancer cells (**C**) using siRARγ. Cells transfected with scrambled siRNA were used as controls. Treatment of siRARγ-transfected cells with 4-[(5,6,7,8-tetrahydro-5,5,8,8-tetramethyl-2-naphthyl)carboxamido]benzoic acid (Am580) (100 nM) in DMSO (0.01% final concentration) further reduced proliferation of MCF-10A and MCF-7 cells, which express RARα, but did not reduce that of the RARα-negative MDA-MB-231 cells. **D**) Relative to c-Myc expression in MCF-10A cells, that in MCF-7 and MDA-MB-231 cells was 38- and 55-fold higher, respectively, according to quantitative PCR. Results shown are representative of two independent experiments.

In order to determine if the pro-proliferative effect of RARγ in the Myc-expressing mouse mammary tumor cells is also present in human mammary cells, *RARγ *was knocked-down with siRNAs in immortalized non-tumorigenic human MCF-10A cells, estrogen receptor (ER^+^) MCF-7 cells and triple negative (ER^-^/progesterone receptor^-^/HER2^-^) MDA-MB-231 breast cancer cells. As shown in Figure 3A-C, the proliferation of all three cell lines was significantly impaired by *RARγ *knockdown. Treatment with 200 nM Am580 enhanced the anti-proliferative effect exhibited by *RARγ *knockdown in the MCF-10A and MCF-7 cell lines but not in the MDA-MB-231 cells. The lack of an effect in MDA-MB-231 is most likely due to the low expression level of *RARα *in these cells [59]. Although, *c-Myc *expression was low in MCF-10A cells and high in MCF-7 and MDA-MB-231 cells) (Figure 3D), this difference did not impinge on the changes in the rate of proliferation induced by modulation of *RARγ *expression.

**Figure 4:**
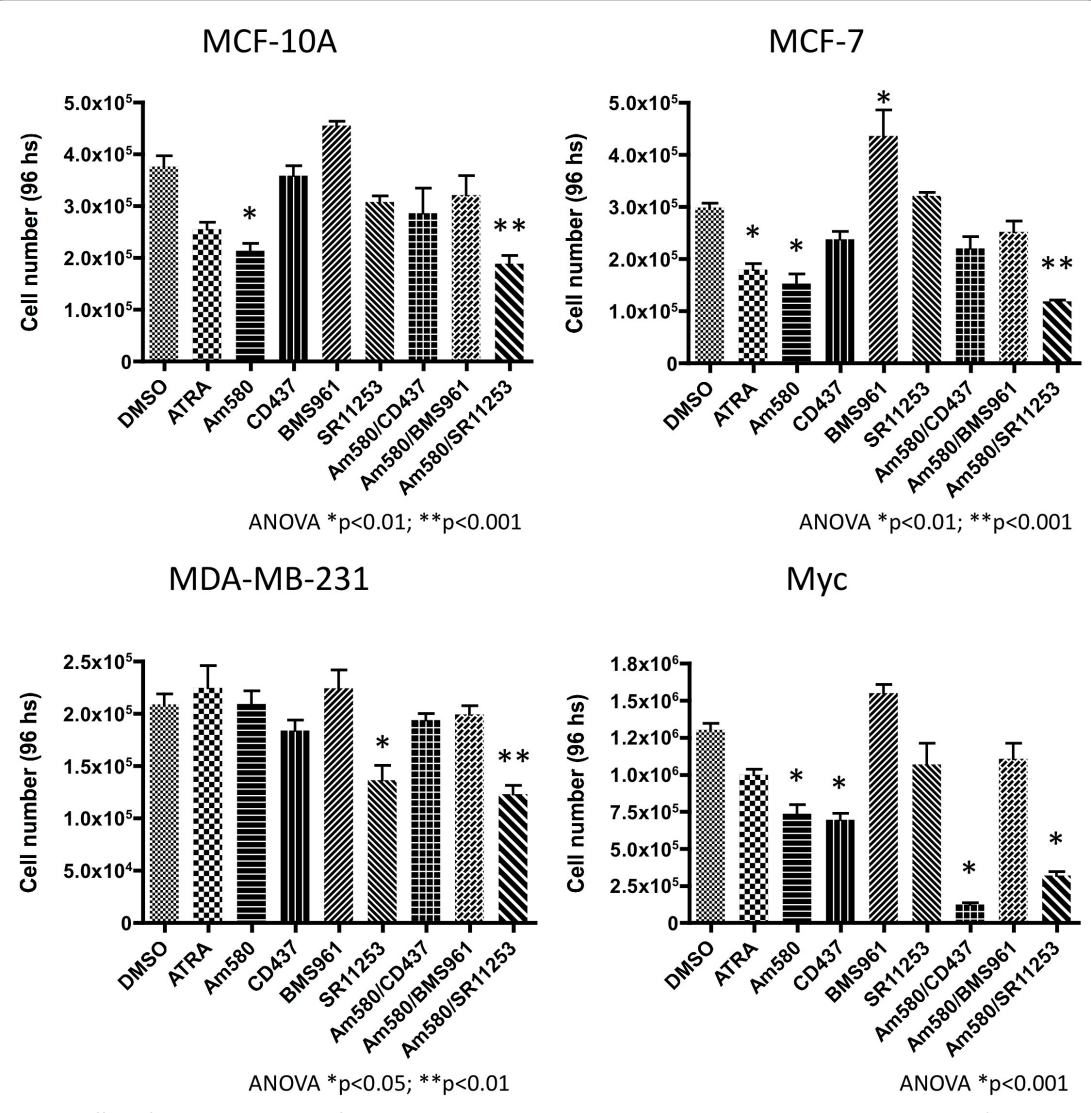
**Effect of retinoids on cell proliferation**. Human MCF-10A, MCF-7, MDA-MB-231 and mouse Myc cells were treated for 96 h with 0.01% DMSO as control, 1 uM All-trans retinoic acid (ATRA) (retinoic acid receptor, RAR, pan-agonist), 200 nM Am580 (RARα agonist), 100 nM CD437 (RARγ and β agonist), 30 nM BMS961 (RARγ agonist), 200 nM SR11253 (RARγ antagonist), and combinations of 4-[(5,6,7,8-tetrahydro-5,5,8,8-tetramethyl-2-naphthyl)carboxamido]benzoic acid (Am580) with CD437, BMS961 and SR11235 at the same concentrations. Cells were counted at the end of the experiment. Results shown are representative of two independent experiments and confirmed by MTS (3-(4,5-dimethylthiazol-2-yl)-5-(3-carboxymethoxyphenyl)-2-(4-sulfophenyl)-2H-tetrazolium) cell viability assays (not shown).

We treated the three human cell lines and Myc-expressing mouse mammary cells with RARγ agonist BMS961, RARγ/β agonist CD347 and RARγ antagonist SR11253 alone, or in combination with ATRA or Am580. Similarly to the results obtained using *RARγ *knockdown (Figure 3), co-treatment of these cell lines, including the mouse Myc cells, with the RARγ antagonist SR11253 and RARα agonist Am580, resulted in strong growth inhibition (Figure 4). As expected, Am580-inhibited MCF-10A- and MCF-7-cell growth (Figure 4, upper panels) were growth-inhibited by Am580, whereas RARγ agonist BMS961 increased their proliferation. Antagonist SR11253 inhibited the proliferation of MDA-MB-231 cells (Figure 4, lower left panel), which was not further inhibited by combination with Am580, as was expected due to their low expression of RARα. The general pattern of responses of the Myc mouse cells to treatment was similar to the human cells, except that the CD437/AHPN (RARγ/β agonist) and its combination with Am580 impaired cell proliferation most effectively (Figure 4, lower right panel). These results show that the pro-proliferative effect of RARγ is ligand-dependent and more importantly, that it can be targeted by RARγ-specific antagonists such as SR11253, alone or in combination with Am580.

**Figure 5:**
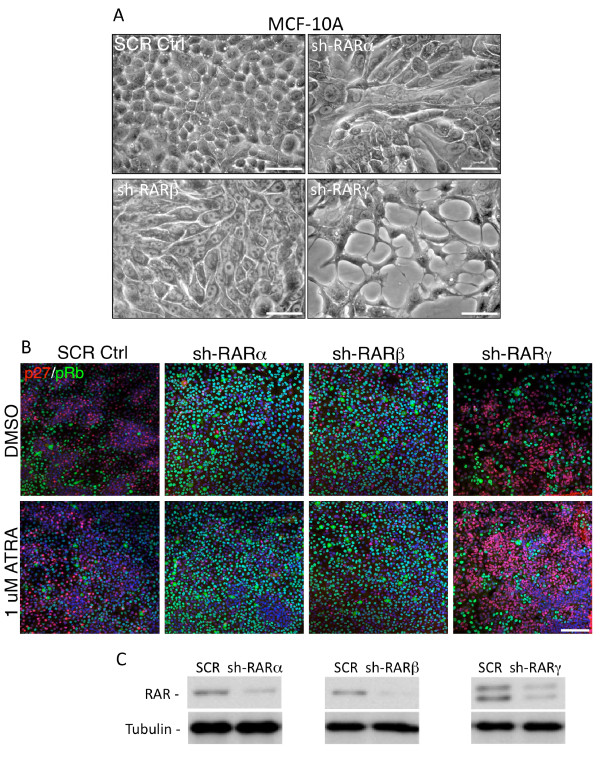
**Down-regulation of retinoic acid receptor (RAR) isotypes in immortalized normal human mammary MCF-10A cells using shRNA affects morphology and response to All-trans retinoic acid (ATRA)**. **A**) Phase-contrast micrographs of MCF-10A cells transfected with shRNA against *RARα*, *RARβ *or *RARγ *show distinct effects on cell morphology compared to nontransfected control cells. Equal numbers of transfected and control cells were seeded and then photographed after 48 h; bar = 20 μm. **B**) Confocal micrographs showing effects on MCF-10A cell proliferation as determined by staining for cell-cycle arrest marker p27 (red) and proliferation marker pRB (green) after transfection with shRARα, shRARβ, shRARγ or scrambled control (SCR) in the presence or absence of 1 uM ATRA; bar = 100 μm. **C**) Immunoblotting with isotype-selective antibodies illustrates silencing achieved with individual shRNAs. Tubulin was used as the loading control. Results shown are representative of three independent experiments.

To further examine the specific roles of the RAR isotypes, each one (α, β or γ) was individually knocked down in MCF-10A cells using specific shRNAs (Figure 5C). Down-regulation of *RARα *or *RARβ *altered the cellular morphology from a more cobblestone-like structure to that of a more spindle-like shape (Figure 5A), suggesting that both isotypes could participate in control of epithelial to mesenchymal transition (EMT). In contrast, down-regulation of *RARγ *impaired cell survival, with cells showing a progressively increasing unhealthy star-like morphology (Figure 5A, right panel). Confocal immunofluorescence analysis using pRb (green) as a surrogate marker for proliferation and p27 (red) for cell-cycle arrest, indicated that knocking down *RARα *or *RARβ *increased proliferation (increased pRb expression), whereas knocking down *RARγ *promoted cell-cycle arrest (increased p27 expression). The p27 levels increased further when shRARγ-transfected cells were treated with 1 uM ATRA for 48 h (Figure 5B), suggesting that changing the ratio of RARα or β to RARγ affects the cellular response to ATRA.

### Effects of RARα activation by Am580 on tumor latency, growth and lung metastasis

**Figure 6:**
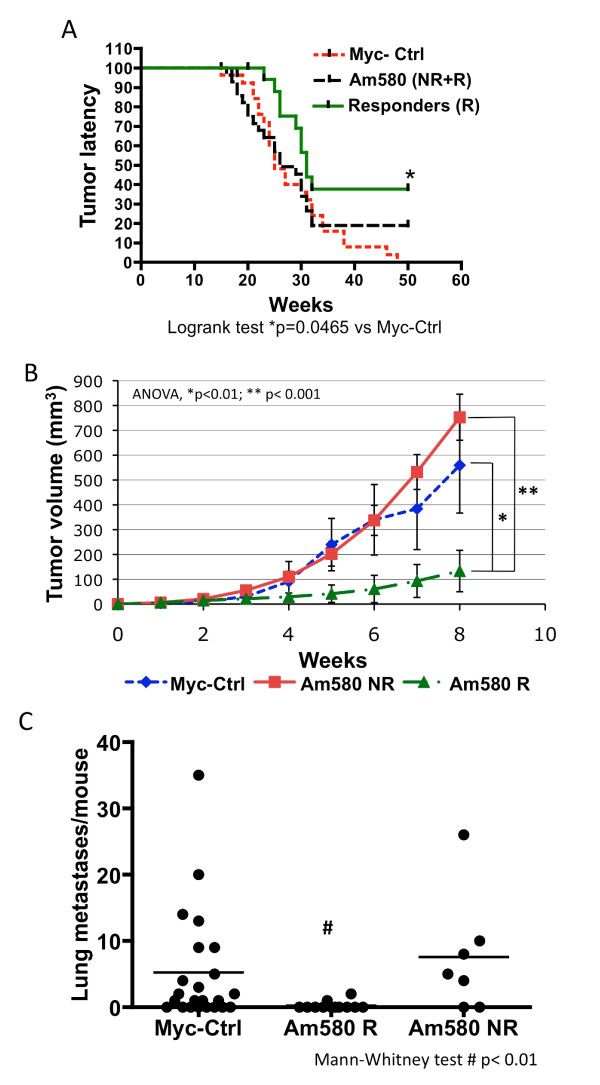
**Effect of Am580 treatment on mammary tumor development, tumor growth and metastatic dissemination in MMTV-Myc mice**. Groups of 30 uniparous MMTV-Myc female mice were given 4-[(5,6,7,8-tetrahydro-5,5,8,8-tetramethyl-2-naphthyl)carboxamido]benzoic acid (Am580) (0.3 mg/Kg/day) in the diet or control diet for up to 50 weeks. **A**) Time to palpable tumor development was recorded to determine percent of mice with tumor-free survival over time. Kaplan-Meier analysis of the data indicated that numbers of mice with extended latency were not significantly different between treated (Am580 (NR+R)) and untreated control (Myc-Ctrl) groups. However, separation of data from the treated group at week 50 on the basis of positive response (Am580 R) or nonresponse (Am580 NR) to Am580 dosing, and reanalysis indicated that the percent of Am580 R mice responding to Am580 as evidenced by the increased tumor latency, from the total Am580 (NR+R) group was significantly higher than the percent of Myc-Ctrl mice (log-rank test, *P *= 0.0456). **B**) Tumor growth was recorded by measuring the two main diameters weekly over an 8-week period beginning when tumors were palpable, and volumes were calculated as described in Methods. Analysis of individual tumor growth per mouse indicated two distinct populations in the Am580 treatment group on the basis of tumor volume, namely responders (Am580 R) and nonresponders (Am580 NR). **C**) Effect of Am580 on metastatic dissemination to lungs in the Myc-Ctrl, Am580 R and Am580 NR experimental groups. The number of overt metastases per mouse lung was quantified under a Nikon stereoscope and plotted. The statistical significance of the results was determined using the Mann-Whitney nonparametric test.

Based on our prior work [39] and the above results, we reasoned that use of a RARα-selective agonist might circumvent the pro-proliferative and pRb repressive effects induced by activation of RARγ [11] and thereby prevent tumor development. To accomplish this goal, we selected the RARα agonist Am580 [60], which was reported to have 10-fold higher binding affinity for RARα than RARβ and no detectable affinity for RARγ [40]. Uniparous, 15-week-old MMTV-Myc female mice, 30 per group, were fed standard diet containing Am580 or the standard diet alone (Myc-Ctrl group) and palpated twice weekly for the appearance of tumors. Tumors were first noted at week 16 in both experimental groups (control diet and Am580 diet). At week 50, no significant difference in tumor development was observed between the two groups analyzed by Kaplan-Meier plot. At that time, 80% of the Am580-treated mice and 100% of the control mice had tumors (Figure 6A, Myc-Ctrl vs. Am580-treated; NR+R, where NR are Am580 non-responders, and R are Am580 responders). Analysis of the tumor growth rates after initial tumor detection revealed two distinct sub-populations in the Am580-treatment group, those with fast-growing tumors (Figure 6B, Am580 non-responders denoted by squares) and those with slow-growing tumors (Figure 6B, Am580-responders denoted by triangles). Of 27 treated mice available for evaluation at week 50, 17 (63%) responded to Am580 treatment with 90% reduction in tumor size relative to the untreated controls, while 10 (37%) did not show a tumor size reduction (non-responders) (Figure 6B). Comparison of the Am580 responders to the Myc-Ctrl group by Kaplan-Meier analysis showed that tumor latency in the responders was significantly extended (*P *= 0.0465, log-rank test, Am580 R vs. Myc-Ctrl), with 35% of the treated Am580-responding mice not developing tumors at 50 weeks (Figure 6B).

Metastatic dissemination of tumor cells is the ultimate cause of death in breast cancer with the lungs being one of the major target organs for metastasis to occur [61]. Analysis of lung metastasis incidence and number of metastatic lesions per mouse revealed that, compared to untreated mice (Myc-Ctrl) of which 66.6% developed lung metastases, only 16.6% of the Am580-responsive mice had metastases. The incidence of metastases in Am580 non-responsive mice was 71.4%, whereas compared to this group, Am580 responders had 36.8% metastasis incidence. The number of metastases per responder mouse was also reduced by Am580 (Figure 6C). Together these results demonstrate the ability of Am580 to reduce tumor growth and aggressiveness in the MMTV-Myc tumor model.

### Difference in Am580 response is related to the RARα/RARγ balance in tumors

Because the MMTV-Myc mouse is an inbred population, we did not expect the difference in response to Am580 to be host-dependent. However, to rule it out formally, two tumors from each Am580 treatment group were excised, minced and then inoculated into the mammary fat pads of separate groups of syngenic FVB females, which have the same genetic background of the MMTV-Myc transgenic model. Inoculated mice were fed the diet containing Am580 for 20 days. The transplanted tumors recapitulated the individual patterns of response and non-response of the original tumor (Additional file 2, Figure S2) indicating that the response to Am580 was independent of the host and intrinsic to the tumor.

**Figure 7:**
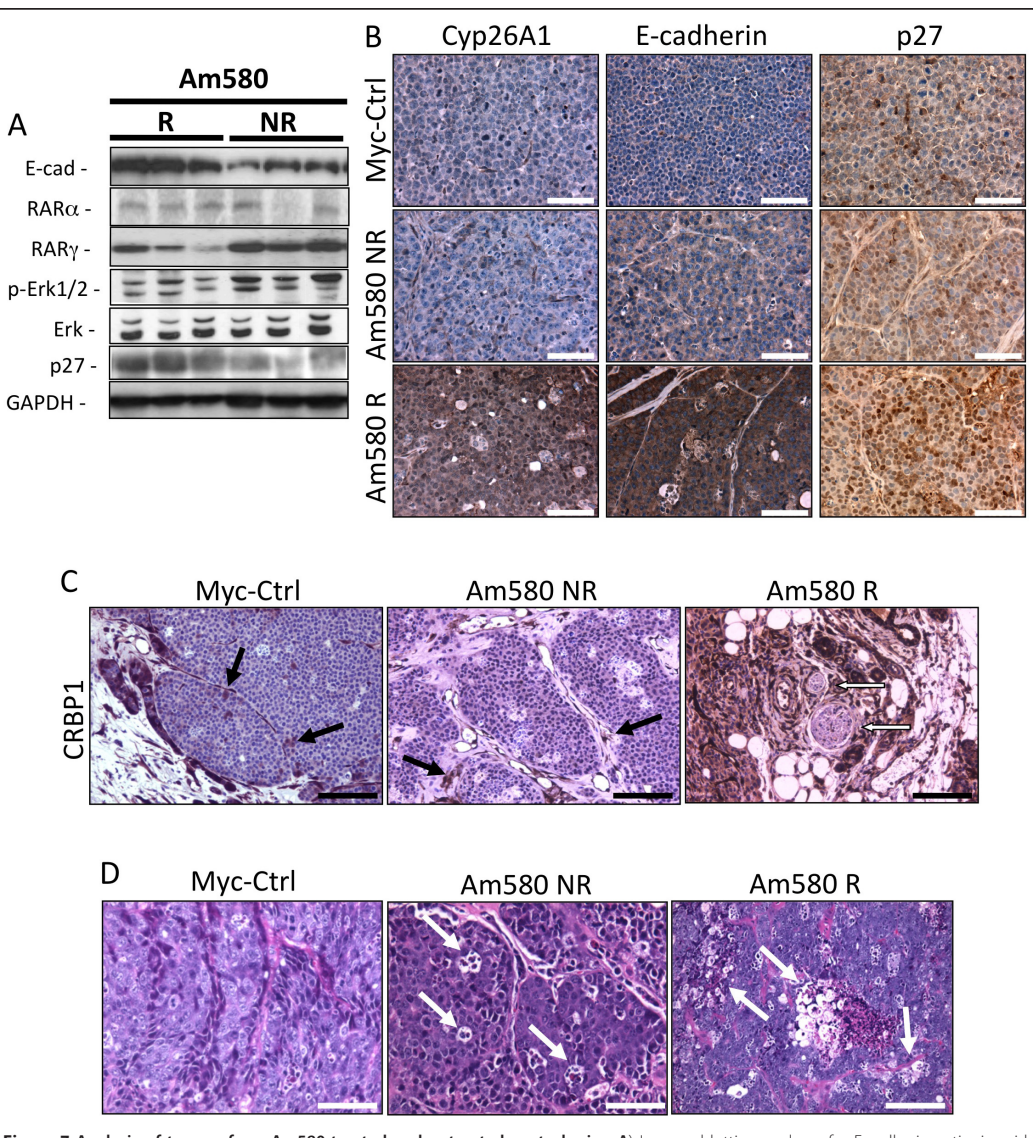
**Analysis of tumors from Am580-treated and untreated control mice**. **A**) Immunoblotting analyses for E-cadherin, retinoic acid receptor (RAR)α, RARγ, phosphorylated Erk (activation) and p27 levels of 4-[(5,6,7,8-tetrahydro-5,5,8,8-tetramethyl-2-naphthyl)carboxamido]benzoic acid (Am580)-responsive (Am580 R) and -nonresponsive (Am580 NR) tumors (three representatives from each group of five tumors shown). Glyceraldehyde-3-phosphate dehydrogenase (GAPDH) was used as a loading control. **B**) Immunohistochemical analyses of RARα-responsive genes Cyp26A, E-cadherin and p27 (brown staining) in sections from (Am580 R, Am580 NR and Myc-Ctrl tumors; scale bar = 200 μm. **C**) Immunohistochemical analysis for CRBP1 expression in tumor sections indicates that tumors from Am580 R mice expressed CRBP1, whereas only stromal cells in tumor sections from the Am580 NR and Myc-Ctrl mice expressed CRBP1 (black arrows). Am580 R tumor sections had small nodules of CRBP1-negative cells (white arrows); bar = 400 μm. **D**) H & E staining of a Myc-Ctrl tumor section reveals an invasive carcinoma with very few necrotic areas, whereas a section from an Am580 NR tumor displays an increase in frequency of necrosis (white arrows; bar = 200 μm) and that from an Am580 R tumor displays larger and more necrotic areas (white arrows; bar = 400 μm). Sections shown are representative of five replicates. Immunohistochemical analysis was done on sections from eight tumors per experimental group.

**Figure 8:**
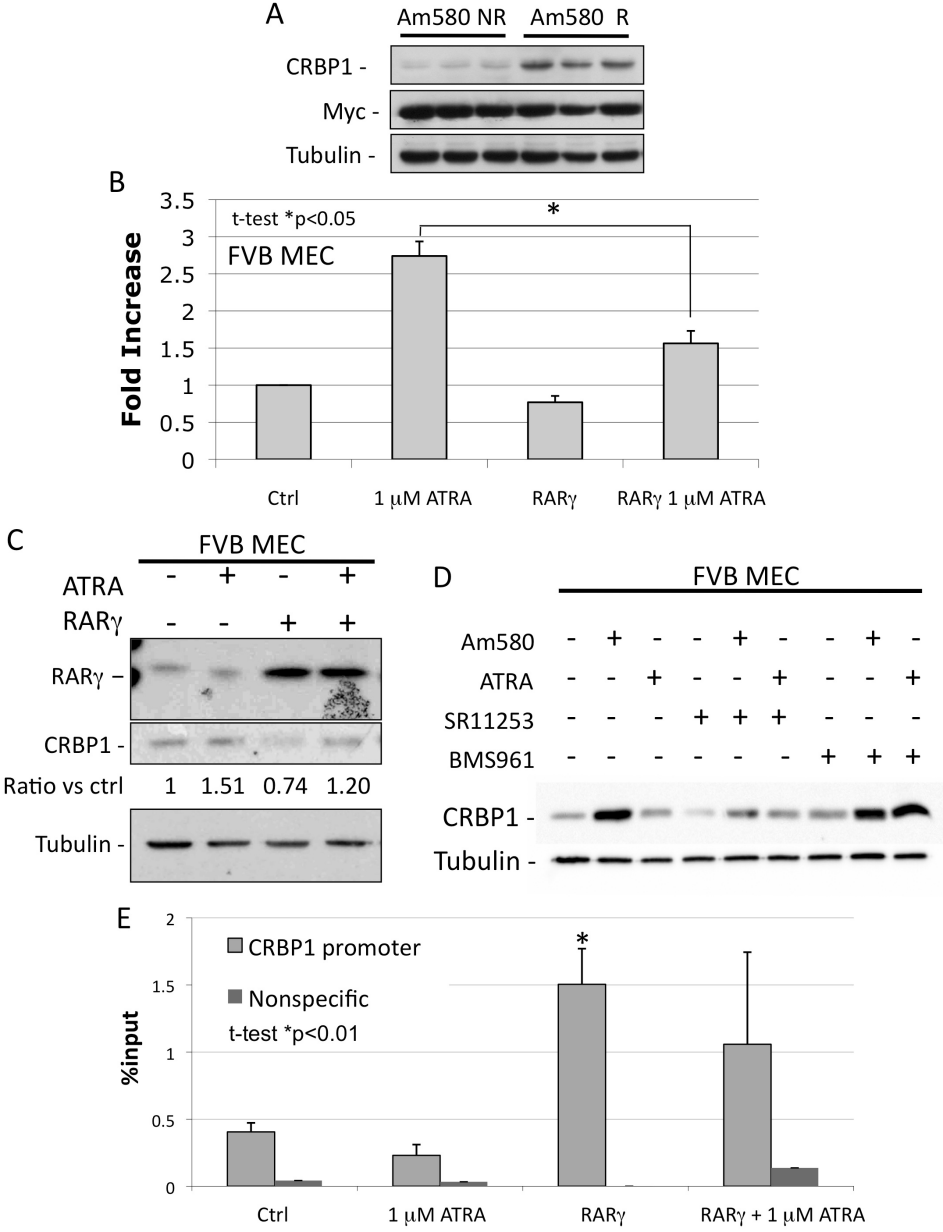
**CRBP1 is a downstream target of retinoic acid receptor (RAR)γ**. **A**) Immunoblotting analysis of CRBP1 protein in tumor lysates from 4-[(5,6,7,8-tetrahydro-5,5,8,8-tetramethyl-2-naphthyl)carboxamido]benzoic acid (Am580)-responsive (Am580 R) and Am580-nonresponsive (Am580 NR) tumors. Results from three representative tumors from each experimental group are shown. Tubulin was used as a loading control. **B**) and **C**) Quantitative (Q)-PCR analysis of CRBP1 gene expression in normal mammary epithelial cells obtained from FVB mice transfected with *RARγ *or vector control and treated with 1 uM All-trans retinoic acid (ATRA) or vehicle control for 48 h. **D**) Primary mammary epithelial cells obtained from a normal FVB female mouse were isolated and used to generate a primary culture that was treated with each retinoid (100 nM Am580, 1 uM ATRA, 100 nMSR11253 and 60 nM BMS961) for 72 h., CRBP1 protein levels were determined by western blot analysis. Results shown are representative of three independent experiments. **E**) Chip analysis of lysates from normal FVB mammary epithelial cells treated or not with 1 μM ATRA for 48 h to examine interaction of RARγ with the *CRBP1 *gene promoter. Results presented are representative of three independent experiments done in triplicate.

We, therefore, tested whether an imbalance in RAR isotype expression defined the response to Am580 treatment. Analysis of RAR isotype protein levels showed that Am580 NR tumors expressed higher RARγ protein levels than Am580 R tumors (Figure 7A). To confirm that high expression of RARγ correlated with lack of responsiveness to Am580, sections from untreated (control) and treated responsive and nonresponsive tumors were examined for protein levels of RARα target genes by western blotting and immunohistochemistry. As shown in Figure 7A, tumor lysates of Am580-responsive mice treated with Am580 exhibited increased levels of growth arrest (p27) and differentiation (E-cadherin) markers compared to the Am580-nonresponsive tumors. Tumor lysates of Am580-treated nonresponsive mice also had increased levels of p-Erk1/2 (Figure 7A), which also suggests an increased proliferative response. RARα protein levels were low in both Am580 treatment groups, whereas RARγ was much higher in the Am580 non-responsive tumors. Immunohistochemistry of tumor sections (Figure 7B) confirmed the western blot results by showing enhanced levels of E-cadherin and p27 in the Am580 responsive tumors and also revealed, that the RARα target gene involved in ATRA catabolism, cytosolic Cyp26A1, was strongly induced in these tumors compared to the Am580 nonresponsive and Myc-Ctrl tumors. Both immunohistochemistry (Figure 7C) and western blotting (Figure 8A) showed that Am580 treatment induced robust CRBP1 expression exclusively in the responsive tumors. The only CRBP1-positive cells detected in the untreated control and nonresponsive tumors were host stromal cells (black arrows in Figure 7C and data not shown). Interestingly, the responsive tumors showed focal outgrowths negative for CRBP1 staining, which may correspond to nonresponsive precursors (white arrows in Figure 7C). Despite induction of several differentiation markers, no overt morphological signs of tissue differentiation, such as the acinar-like structures observed in the three-dimensional cultures (Figure 1E), were detected in these tumor sections. However, scattered necrotic areas in both nonresponsive and responsive tumor sections were observed and found to be bigger and more prevalent in the responsive sections (Figure 7D, white arrows).

### RARγ down-regulates CRBP1 expression by direct transcriptional repression

Since RARγ and CRBP1 expression levels were inversely regulated in c-Myc-expressing tumors (Figure 1B), as shown by up-regulation of CRBP1 protein by RARγ inhibition [39] and (Figure 1D), we explored the possibility that ligand-free RARγ could transcriptionally repress CRBP1. We ectopically expressed *RARγ *in primary mammary epithelial cells isolated from FVB mice and analyzed its effect on CRBP1 expression (mRNA) and protein levels (Figure 8B and 8C, respectively) and found a slight down-regulation in *CRBP1 *levels. ATRA treatment strongly induced *CRBP1 *expression at mRNA (Figure 8B) and, to a lesser degree, protein levels (Figure 8C). It is possible that the CRBP1 protein level continues to accumulate beyond the 24 h shown here, as was shown in rat Sertoli cells in which the half-life of CRBP1 was calculated to be 48 h [62].

To test the regulation of CRBP1 in mammary epithelial cells by retinoids, primary mammary epithelium from three 9-week-old FVB females, was treated for 3 days with 100 nM Am580, 1 uM ATRA, 100 nM SR11253, 60 nM BMS961, or their combination and CRBP1 levels were evaluated by western blot analysis (Figure 8D). CRBP1 protein was increased by RARα agonist Am580 alone or with RARγ agonist BMS961 (Figure 2 second and eighth lanes) and by RAR pan-agonist ATRA plus BMS961 (Figure 2 ninth lane) but reduced by RARγ antagonist SR11253. No major changes in CRBP1 expression level were observed in cells treated with ATRA, Am580 plus SR11253, ATRA plus SR11253, or BMS961. These results show that in contrast to Myc-expressing cells, CRBP1 protein levels are reduced in normal mammary epithelium when RARγ transcriptional activation is blocked, suggesting that oncogenic stress changes the RARγ function, possibly through changes in the RARγ-associated transcriptional complex, additional studies are required to elucidate these differences.

Although we did not examine the composition of the transcriptional complex, using ChiP assays we showed that over-expression of *RARγ *in normal mammary cells led to an enrichment of RARγ promoter (Figure 8E); whereas treatment of these cells with ATRA led to a slight *CRBP1 *reduction in *CRBP1 *promoter-associated RARγ and re-expression of *CRBP1 *(Figure 8B-D). These results suggest a repressive role for RARγ. Because ATRA activates all three RAR isotypes, we propose that in normal cells, activated RARα competes with RARγ for binding to the RARE in the *CRBP1 *promoter. Under the influence of over-expressed c-Myc, the ratio of RARα/RARγ changes profoundly in favor of RARγ, thus limiting the ability of RARα to displace RARγ from this promoter.

## Discussion

Our data show that c-Myc over-expression in mammary epithelial cells alters the RARα -β/RARγ balance through RARγ up-regulation. The relative increase in RARγ isotype expression relative to the α and β isotypes correlates with tumor progression and lack of expression of RARα target genes involved in cell-cycle arrest and differentiation. In contrast, a shift towards RARα activation by the selective RARα agonist Am580 induces a tumor-inhibiting response in MMTV-Myc mice. Of the Am580-treated mice, approximately 63% responded to Am580 treatment by showing an anti-tumor effect with decreased tumor growth rates and lower incidence and number of lung metastases, while the rest (37%) did not respond to Am580. Only the responders displayed reduced levels of RARγ protein in their tumors, accompanied by increased expression of RARα target genes.

Collectively, several specific results obtained here identify RARγ as a pro-oncogenic RAR isotype. First, antagonism of RARγ transactivation by pharmacologic levels of its antagonist SR11253 dose-dependently halted the rapid growth of cells that over-expressed the oncogene Myc. Second, RARγ knockdown in MMTV-Myc cells, enabled RARα-selective Am580 to more effectively inhibit cell growth and to increase levels of the RARα target gene, *CRBP1*. Moreover, *CRBP1 *expression was higher in MMTV-Myc tumors in mice that responded to Am580 treatment. This result is important because we previously reported that re-expression of CRBP1 was associated with impaired tumor progression [42,43].

CRBP1 expression also decreased after normal mammary epithelial cells were ectopically transfected with *RARγ*, whereas specific pharmacologic activation of RARγ increased CRBP1 expression. The fact that in normal mammary epithelia RARγ-mediated repression of CRBP1 expression occurred only in the absence of its agonist, or at low agonist concentration (due to the presence of retinol in the serum), suggests that additional changes such as those induced by such oncogenes as *c-Myc *are required to potentiate the gene repressive function of RARγ in the presence of ligand.

In addition to the role of RARγ in Myc-mouse mammary epithelial cells, we documented its pro-oncogenic role in human breast cancer cells lines. Knockdown of RARγ expression in immortalized epithelial MCF-10A (ER^-^, non-tumorigenic), MCF-7 (ER^+^, tumorigenic) and MDA-MB-231 (ER^-^, PR^- ^and HER2^-^,tumorigenic) breast cancer cell lines led to their reduced proliferation. With the exception of MDA-MB-231 cells, proliferation was further reduced by treatment with RARα agonist Am580. The lack of response by MDA-MB-231 cells may most likely be due to their low levels of RARα [59] (also see Figure 3). This reduction in cellular proliferation by the addition of Am580 implies that RARα is the tumor suppressor isotype. This conclusion was further strengthened by the experiments (Figure 5) in which selective down-regulation of RARα or RARβ in MCF-10A cells promoted the appearance of a less differentiated, more proliferative phenotype, while reduction of *RARγ *expression led to cell-cycle arrest and reduced cell survival (Figure 5).

The results from the experiments using a genetic approach to RAR isotype regulation were recapitulated using synthetic RAR isotype-specific retinoids in both human and the mouse cell lines. Individually, a RARα agonist and a RARγ antagonist reduced cell growth and their combination was even more effective. The RARγ agonist significantly induced the growth of MCF7 breast cancer cells. Interestingly, MDA-MB-231 cells, which did not respond to Am580, became sensitized to Am580 by co-treatment with RARγ antagonist SR11253. The combination of Am580 with CD437/AHPN in the Myc cells gave the strongest growth inhibition; we believe that this strong response is probably related to the RAR-independent pro-apoptotic effects described for the CD437 that interestingly include the inhibition of c-Myc expression [63-65]. In this regard Paroni *et al*. [66] have shown that RARα was co-amplified in approximately one third of ERBB2^+ ^human breast cancers. In culture, treating such cells with the ERBB2 inhibitor lapatinib combined with ATRA synergistically inhibited growth, and induced cell differentiation and apoptosis. We previously showed that in transgenic mice bearing MMTV-Neu- and -Wnt1-driven tumors [39], and report here that in mice bearing MMTV-Myc-driven tumors and in human breast cancer cell lines, *RARγ *expression is counterproductive to the anti-cancer effects of ATRA. On this basis, we propose that substituting a RARα-selective or specific agonist for the RAR pan-agonist ATRA should improve the therapeutic response.

Our findings are unique in that they describe a specific role for c-Myc-mediated differential regulation of RAR isotypes in which predominance of RARγ facilitates the pro-oncogenic phenotype. Our results are in agreement with the proliferative role of RARγ in hematopoiesis [3] and hepatocellular carcinoma [38]. We find that in the context of *c-Myc *expression the oncogenic function of RARγ is potentiated. Myc increases *RARγ *expression, leading to changes in the stoichiometry between RAR isotypes. Knockdown of RARγ in MMTV-Myc-derived cancer cells impaired tumor growth in a xenograft model and induced markers of cell-cycle arrest, differentiation and acinar-like morphogenesis in three-dimensional cultures.

Functional specificity of RAR depends on the ability of these isotypes to bind and recruit other co-repressors or co-activators [67]. However, most studies to date have focused on the presence of RARα or the lack of RARβ. Privalsky *et al*. showed that RARγ has low affinity for classical co-repressors associated with nuclear receptors, such as N-CoR or SMRT [68], suggesting a different mechanism of repression would be induced by RARγ. In a recent paper [69], RARγ was shown to control both the association and dissociation of the chromatin repressor Suz12 from transcriptional complexes. Suz12 belongs to the Polycomb Repressive Complex 2 and its up-regulation has been associated with cancer and stem cell compartment maintenance [70,71]. It is possible that oncogenic stress induces an up-regulation of both RARγ and Suz12, promoting an aberrant association between the two proteins leading to abnormal RARγ function and gene expression outcome.

Polycomb Group (PcG) proteins are known to control the recruitment of DNA methyltransferases (DNMT) to target promoters [72] and it is possible that over-expressed RARγ bound to the *CRBP1 *promoter favors this process. This repressive effect of RARγ on *CRBP1 *expression may be an early response to c-Myc oncogenic transformation that reduces *CRBP1 *expression, which later becomes permanent, by promoter hypermethylation [73,74]. In such a scenario RARα may no longer be able to compete for *CRBP1 *promoter occupancy so that cells became insensitive to Am580 treatment. Although, this is an attractive and testable scenario, we can exclude the possibility that the decrease in *CRBP1 *expression caused by RARγ up-regulation is due to the lack of recruitment of transcription activators to *CRBP1 *promoter, suggesting that RARγ is a less efficient transactivator instead of being a strict repressor. Further analysis is required to determine how a shift in the RAR isotypes induced by c-Myc affects co-activators, co-repressors and other regulatory components of the transcriptional complexes.

As a whole, our results suggest that RARγ has an opposite role in tumorigenesis than RARα or RARβ. Oncogenic stress, which deregulates important cell signaling pathways and alters the normal function of nuclear receptors such as RARs, can induce *RARγ *expression and its pro-survival function inducing RARα-β/RARγ imbalance and aberrant repression of RARα-target genes. Pharmacologic activation of RARα, or inhibition of RARγ activity, reduces cancer cell growth *in vitro*, thereby suggesting that a specific RARα agonist would be a more effective method of cancer treatment than an RAR pan-agonist such us ATRA.

## Conclusions

Genetic and pharmacologic approaches in cell culture and *in vivo *allowed us to conclude that maintenance of a proper RARαβ/γ balance is crucial for preserving normal epithelial cell homeostasis. An oncogenic signal initiated by *c-Myc *over-expression increases *RARγ *expression, which perturbs the RARαβ/γ balance in favor of RARγ. The consequence of this change is down-regulation of tumor suppressive genes such as *RARα*, *RARβ *and *CRBP1*, and possibly others. This pro-tumorigenic c-Myc induced effect can be overcome by selective activation of RARα that leads to the re-expression of *CRBP1 *and reversion of the malignant phenotype. Elucidating the mechanism through which RAR isotypes are involved in c-Myc tumorigenesis is clinically relevant since *c-Myc *expression is altered in 25% of human breast cancers [75-78]. The combined results of the present study alongside those previously published [39] support the use of a RARα-selective agonist and/or RARγ-selective antagonist as a therapeutic approach for a subset of patients. We propose that determining the balance of RAR isotypes in cancer tissues compared to normal ones will improve tumor classification and will identify those tumors in which RAR isotype-selective intervention would be of benefit to the patients.

## Abbreviations

Am580: 4-[(5,6,7,8-tetrahydro-5,5,8,8-tetramethyl-2-naphthyl)carboxamido]benzoic acid; ATCC: American Type Culture Collection; ATRA: *All-trans *retinoic acid; BSA: bovine serum albumin; CRBP1: cellular retinol-binding protein 1; DAB: 3,3'-diaminobenzidine; DMEM: Dulbecco's modified Eagle's medium; DMSO: dimethyl sulfoxide; EGF: epithelial growth factor; EMT: epithelial to mesenchymal transition; ER: estrogen receptor; FBS: fetal bovine serum; FVB: Friend Virus B wild type mice; GAPDH: glyceraldehyde-3-phosphate dehydrogenase; H & E: haematoxylin and eosin; IACUC: Institutional Animal Care and Use Committee; IgG: immunoglobulin G; MEC: mammary epithelial cells; MMTV: mouse mammary tumor virus; NR: nonresponders; PBS: phosphate buffered saline; PCR: polymerase chain reaction; R: responders; RA: retinoic acid; RAR: retinoic acid receptor; RARE: retinoic acid response element; RIPA: radioimmunoprecipitation assay; RXR: retinoid × receptor; SDS: sodium dodecyl sulfate.

## Competing interests

The authors declare that they have no competing interests.

## Authors' contributions

AB generated, assembled and analyzed the data on gene expression, protein levels and ChIP analysis. SPB generated and genotyped the transgenic mice required for the *in vivo *experiments, and collected and analyzed the *in vivo *data. YL conducted the *in vivo *treatments, collected the spontaneous tumor development data and performed the immunohistochemical analysis of the tumors. AG participated in the collection and analysis of the *in vitro *an *in vivo *data. AMJ conducted and assembled the *in vitro *proliferation assays. MID supplied RARγ agonist and antagonist, technical assistance in the compound used and critical editing of the manuscript. EFF conceived and designed the study, collected and assembled the data, performed data analysis and wrote the manuscript. All the authors participated in the edition of the manuscript and have read and approved the final manuscript.

## Supplementary Material

Additional file 1 **Figure S1: Gene expression controls**. **A**) Quantitative (Q)-PCR showing expression levels of human c-Myc in FVB mammary epithelial cells after transfection with pcDNA3 vector control (FVB-Ctrl) or with pcDNA3-h-*c-Myc *(FVB-Myc). **B**) Q-PCR showing retinoic acid receptor (*RAR*) γ expression is knocked-down by shRARγ in Myc cells. Click here for file

Additional file 2 **Figure S2: Am580 NR and Am580 R xenograft responses to Am580**. Because the mouse mammary tumor virus (MMTV) promoter can be expressed in some cells of the immune system, it was important to determine whether the MMTV-Myc xenograft host FVB animal influenced response to 4-[(5,6,7,8-tetrahydro-5,5,8,8-tetramethyl-2-naphthyl)carboxamido]benzoic acid (Am580) treatment. Therefore, tumor cells obtained from two Am580-nonresponsive (NR1 and NR2) and two Am580-responsive (R1 and R2) xenografts that had been obtained from Am580-treated MMTV-Myc mice were injected into the abdominal mammary fat pad of FVB syngenic females (n = 10, analysis of variance *P*<0.001) and tumor growth was monitored. Both Am580 R and Am580 NR xenografts retained their respective slow and fast tumor growth rates in their FVB hosts as those observed in tumors from the original transgenic groups indicating that growth behavior was intrinsic to the tumors, and not associated with an immunologic response by the host mice. Click here for file
