# Supplementary figures and images for: Reversal by RARα agonist Am580 of c-Myc-induced imbalance in RARα/RARγ expression during MMTV-Myc tumorigenesis
Source: Breast Cancer Res. 2012 Aug 24;14(4):R121. doi: 10.1186/bcr3247 (PMC3680916; doi:10.1186/bcr3247)

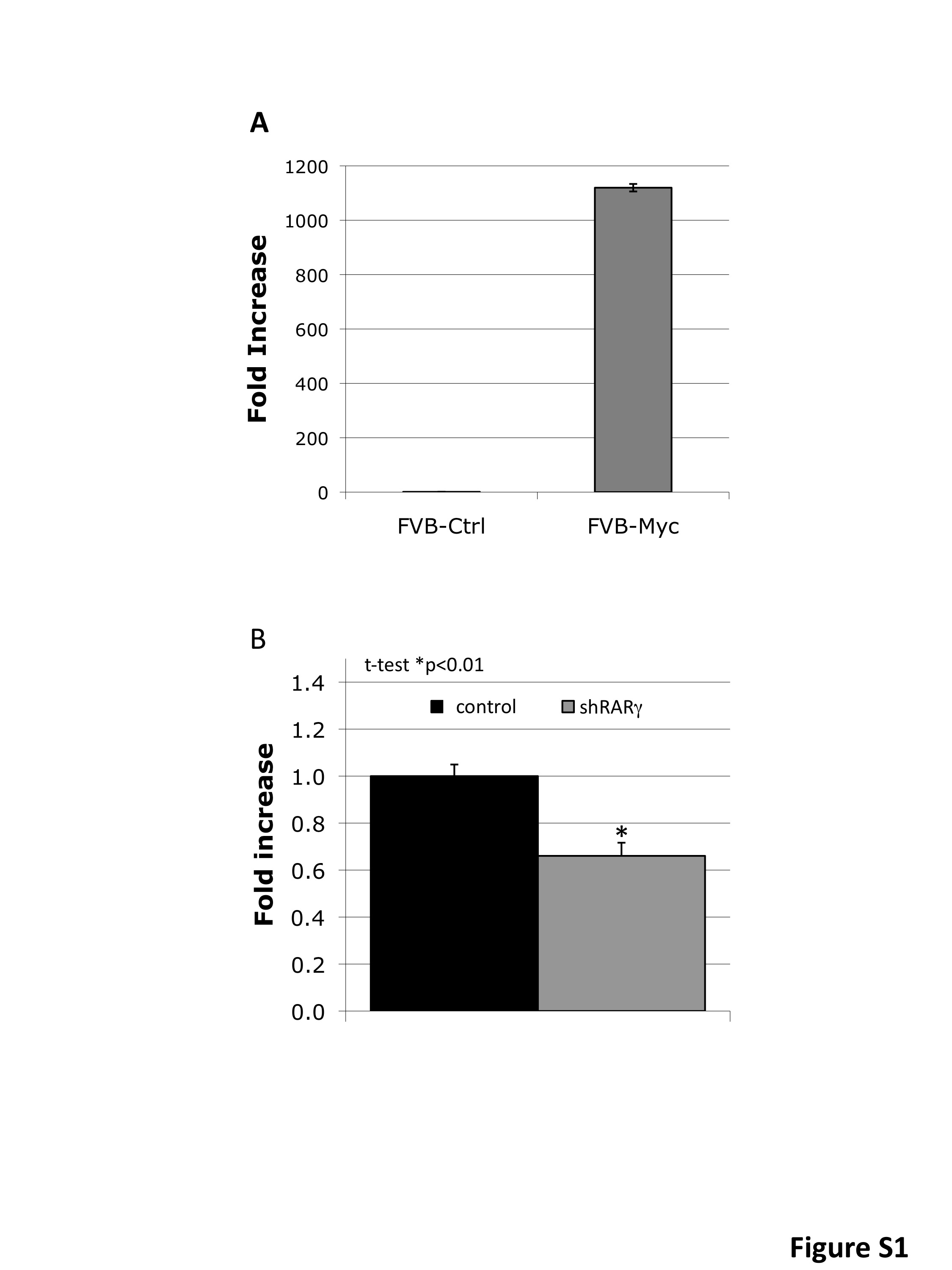

Supplement: Additional file 1 — Figure S1: Gene expression controls. A) Quantitative (Q)-PCR showing expression levels of human c-Myc in FVB mammary epithelial cells after transfection with pcDNA3 vector control (FVB-Ctrl) or with pcDNA3-h-c-Myc (FVB-Myc). B) Q-PCR showing retinoic acid receptor (RAR) γ expression is knocked-down by shRARγ in Myc cells. [file bcr3247-S1.JPEG]

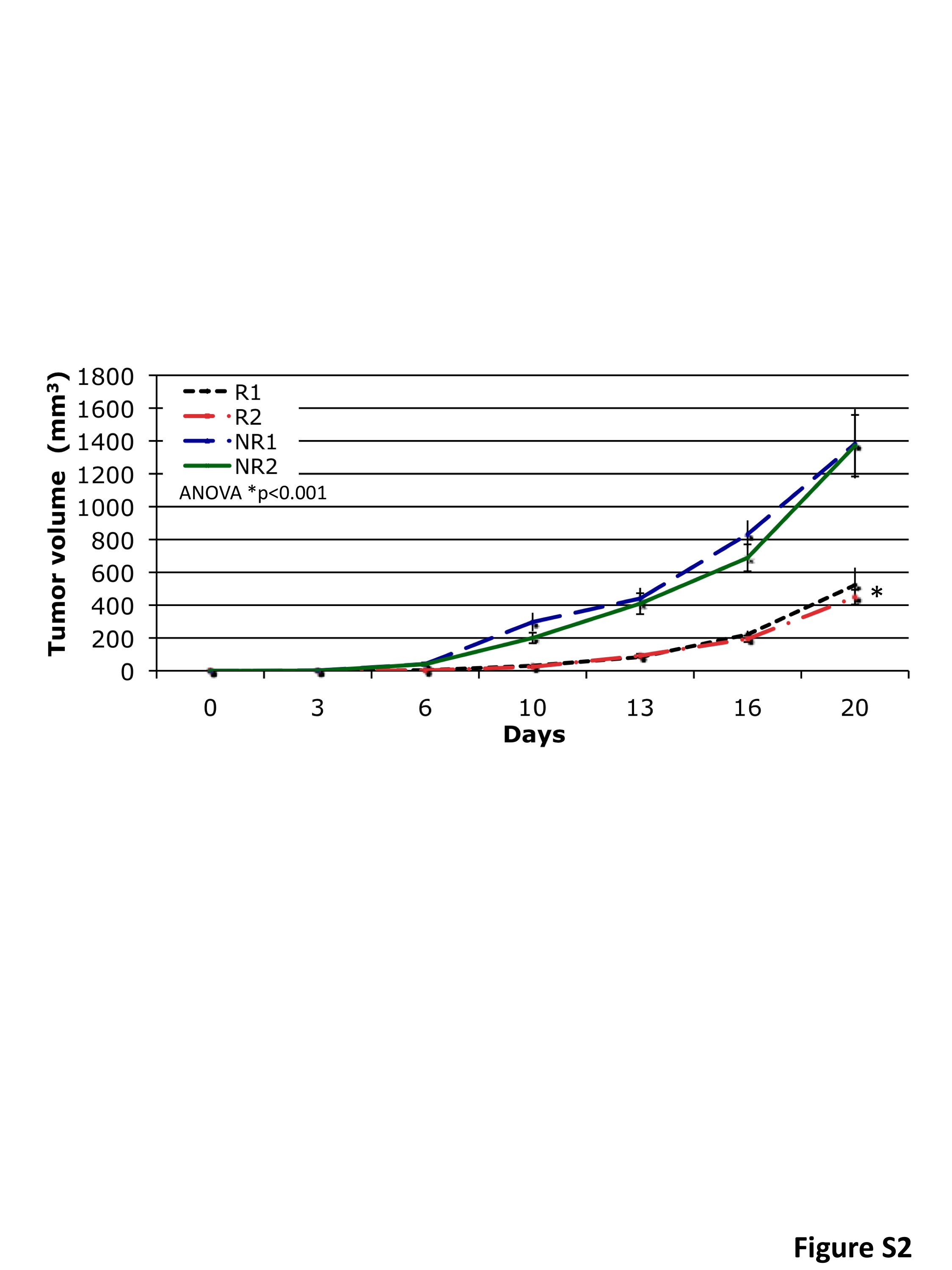

Supplement: Additional file 2 — Figure S2: Am580 NR and Am580 R xenograft responses to Am580. Because the mouse mammary tumor virus (MMTV) promoter can be expressed in some cells of the immune system, it was important to determine whether the MMTV-Myc xenograft host FVB animal influenced response to 4-[(5,6,7,8-tetrahydro-5,5,8,8-tetramethyl-2-naphthyl)carboxamido]benzoic acid (Am580) treatment. Therefore, tumor cells obtained from two Am580-nonresponsive (NR1 and NR2) and two Am580-responsive (R1 and R2) xenografts that had been obtained from Am580-treated MMTV-Myc mice were injected into the abdominal mammary fat pad of FVB syngenic females (n = 10, analysis of variance P<0.001) and tumor growth was monitored. Both Am580 R and Am580 NR xenografts retained their respective slow and fast tumor growth rates in their FVB hosts as those observed in tumors from the original transgenic groups indicating that growth behavior was intrinsic to the tumors, and not associated with an immunologic response by the host mice. [file bcr3247-S2.JPEG]
